# An analytical upper bound on the number of loci required for all splits of a species tree to appear in a set of gene trees

**DOI:** 10.1186/s12859-016-1266-4

**Published:** 2016-11-11

**Authors:** Lawrence H. Uricchio, Tandy Warnow, Noah A. Rosenberg

**Affiliations:** 1 Department of Biology, Stanford University, Stanford, CA, USA; 2 Departments of Computer Science & Bioengineering, University of Illinois, Urbana-Champaign, IL, USA

**Keywords:** Bipartitions, Coalescent, Gene trees, Species trees

## Abstract

**Background:**

Many methods for species tree inference require data from a sufficiently large sample of genomic loci in order to produce accurate estimates. However, few studies have attempted to use analytical theory to quantify “sufficiently large”.

**Results:**

Using the multispecies coalescent model, we report a general analytical upper bound on the number of gene trees *n* required such that with probability *q*, each bipartition of a species tree is represented at least once in a set of *n* random gene trees. This bound employs a formula that is straightforward to compute, depends only on the minimum internal branch length of the species tree and the number of taxa, and applies irrespective of the species tree topology. Using simulations, we investigate numerical properties of the bound as well as its accuracy under the multispecies coalescent.

**Conclusions:**

Our results are helpful for conservatively bounding the number of gene trees required by the ASTRAL inference method, and the approach has potential to be extended to bound other properties of gene tree sets under the model.

## Background

The genomic era presents new challenges for phylogenetic inference studies, because evolutionary processes such as incomplete lineage sorting can cause gene trees at different genomic loci to have different topologies. Many modern techniques therefore capitalize on the independent information available at multiple loci in order to inform phylogenetic estimates (e.g. [1–6]).

One family of phylogenetic methods employs “consensus estimation,” in which a set of gene trees on a shared taxon set is used to infer a single consensus species tree that summarizes the information in the input gene tree collection [7, 8]. In a consensus method—and in more general “summary” methods that do not necessarily require the taxon set to be identical across loci—for each of a series of genomic loci, a rooted or unrooted gene tree is first computed, and particular features of the gene tree set are used to compute an estimate of the species tree (e.g. [8–16]).

Consensus and summary methods are often chosen in species tree inference studies because they typically have desirable properties, including computational efficiency, scalability to trees with many taxa, and conceptual simplicity. This latter feature makes such methods suitable for mathematical analyses, many of which have emphasized the property of statistical consistency under a standard model for gene tree evolution, the multispecies coalescent [3]. In a consistent method, as the number of sampled gene trees increases, the probability that the species tree estimate from a random sample of gene trees produced under the model accords with the true species tree topology approaches 1, irrespective of the species tree topology and branch lengths. Many consensus and summary methods have been shown to be consistent under the multispecies coalescent model (e.g. [8, 10, 11, 13–16]), further justifying their applicability in species tree inference problems.

**Fig. 1:**
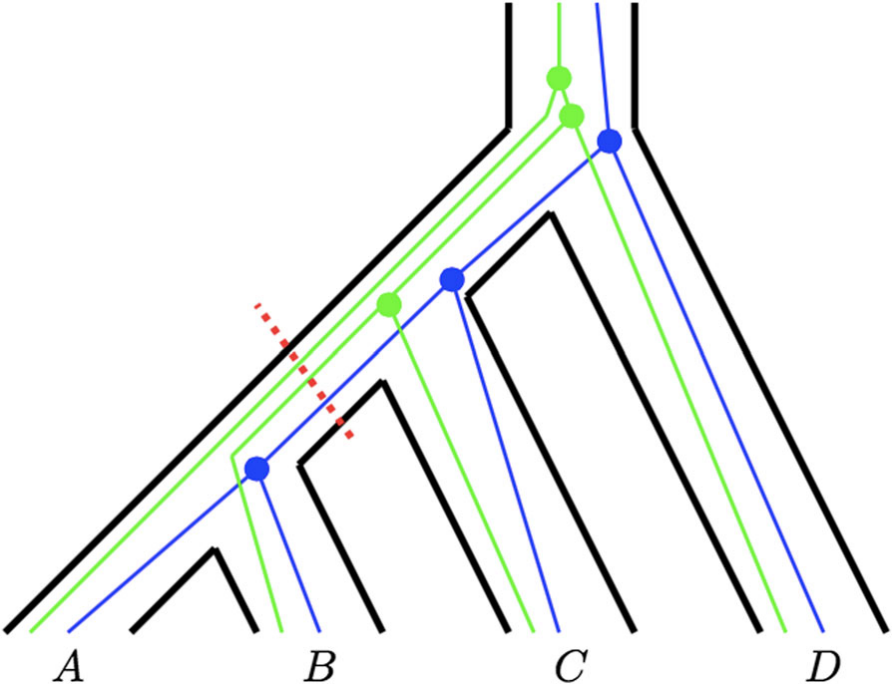
Schematic of a species tree (*black*) and two gene trees (*blue*, *green*). Coalescent events in a gene tree are constrained to occur only once lineages are present in the same population. The red dashed line indicates a species tree bipartition AB |CD, separating species A and B from species C and D. The same bipartition occurs in the blue gene tree; by contrast, the green gene tree does not contain this bipartition, instead containing AD |BC

Mirarab et al. [17] developed one such method: ASTRAL. Given a tree, a *bipartition*, or *split*, corresponds to a cut on one of the branches of the tree, dividing the taxa into two subsets (Fig. 1). Define a gene tree set $\mathcal {G}$ on the same taxon set as the species tree to be a *bipartition cover* of the species tree if for each bipartition in the species tree, at least one gene tree in $\mathcal {G}$ possesses the bipartition. ASTRAL—and the efficiency improvement ASTRAL-II [18]—reports a species tree estimate by searching a space of species trees that draw their bipartitions from a specified input set *X*. Choosing *X* to be the set of bipartitions in $\mathcal {G}$ suffices to ensure that ASTRAL is statistically consistent under the multispecies coalescent model [17], because as increasingly many gene trees are included in $\mathcal {G}$, the probability approaches 1 that each bipartition in the true species tree will appear in at least one gene tree, so that $\mathcal {G}$ will be a bipartition cover with probability approaching 1.

How many gene trees are required so that a random set of gene trees is likely to be a bipartition cover of the species tree? For consistent methods, by definition, asymptotically as the number of gene trees increases without bound, the species tree estimate will be accurate with probability 1. However, relatively few analytical recommendations are available for the number of loci required before the probability is high that specified properties of gene tree sets are achieved [8, 19–21]; in the case of ASTRAL, the consistency proof gives no guidance on the number of gene trees required before $\mathcal {G}$ is likely to be a bipartition cover. In place of an analytical treatment, the speed of convergence of consistent methods might typically be examined by simulation-based evaluations (e.g. [10, 22, 23]); although simulations can provide useful insights into the number of required loci, both because they do not produce provable findings and because their parameter choices are inexhaustive, they can have limited generality.

Here, we produce a general analytical upper bound for the minimal number of gene trees required for a gene tree set to produce with high probability a bipartition cover of the species tree. As a function of the number of taxa in the species tree, a probability threshold, and a single additional parameter describing the species tree branch lengths, we determine an upper bound on the number of loci needed before the bipartition set represented in a collection of gene trees includes—with the specified minimum probability—all bipartitions in the true species tree. We compare the analytical upper bound to values computed using simulations under the multispecies coalescent model. Our approach can potentially assist in obtaining other, similar upper bounds for the number of loci required before other specific features are likely to appear in gene tree collections.

## Results and discussion

### Gene tree discordance and the multispecies coalescent

We begin by briefly reviewing the multispecies coalescent model. Under the model, the genealogical history of orthologous lineages from *k* species is modeled backward in time conditional on a fixed rooted species tree with topology and branch lengths specified. Looking back in time, lineages from a pair of species cannot share common ancestry more recently than the time at which the species share common ancestry (Fig. 1). As a result, conditional on the species tree, not all topologies are equally likely for the gene tree; moreover, a random sample of gene trees that have evolved on the species tree contains information about the species tree topology and branch lengths [24]. In a general treatment of the model, the number of lineages per species is arbitrary, but here we restrict attention to one lineage per species.

Studies of the properties of inference methods applied to sets of gene trees produced under the model can make use of analytical formulas for the probability distribution of gene tree topologies conditional on a species tree [22, 25]. Such formulas employ the species tree topology and branch lengths as parameters, producing a discrete distribution that contains a probability for each possible gene tree topology. This distribution is complex, potentially with significant weight on gene tree topologies that disagree with the species tree, and its properties can differ substantially for species trees with different topologies and different numbers of species [25–28]. In general, under the model, the extent of the disagreement of gene tree topologies with species tree topologies increases as branch lengths in species trees decrease [9, 25], particularly when multiple short branches occur in succession [29].

A key quantity in evaluating gene tree probabilities is a function *g*_*i*,*j*_(*T*) that computes the probability that exactly *i*−*j* coalescent events happen in time *T*, beginning from *i* lineages at time 0 [30]:

1 $$ g_{i,j}(T) = \sum_{k=j}^{i}\frac{e^{-k(k-1)T/2} (2k-1)(-1)^{k-j}j_{(k-1)} \, i_{[k]}}{j! \, (k-j)! \, i_{(k)}},  $$

where *a*_(*k*)_=*a*(*a*+1)…(*a*+*k*−1), *a*_[*k*]_=*a*(*a*−1)…(*a*−*k*+1), and *a*_(0)_=*a*_[0]_=1. *T* is measured in coalescent time units, representing a number of generations normalized by the number of gene copies of a locus present in a population (2*N* for diploids, where *N* is the effective population size measured as a number of individuals).

### Bipartitions

A tree with *k* leaf nodes has 2*k*−3 bipartitions: *k*−3 nontrivial bipartitions in which each of the subsets has at least two leaves, and *k* trivial bipartitions produced from cuts that separate one leaf from the other *k*−1 leaves. The *k* trivial bipartitions appear in every tree topology with a fixed leaf label set; henceforth we assume that bipartitions are nontrivial unless otherwise noted. The number of leaves in the larger of the two leaf subsets of a (nontrivial) bipartition is at most *k*−2. The bipartition separating, for example, taxa A and B from taxa C and D, is annotated AB |CD (Fig. 1).

Consider a species tree and a gene tree—both on the same taxon set—in which one gene tree lineage is sampled per species. We say that a nontrivial bipartition *ϕ* of the species tree is *observed* in the gene tree if for some internal node of the gene tree, a cut on that branch produces the bipartition *ϕ* of the leaf nodes. For a set $\mathcal {G}$ of gene trees, if each of the *k*−3 nontrivial bipartitions of a species tree *S* is observed for at least one gene tree in the set, we say that $\mathcal {G}$ is a *bipartition cover* of *S*.

For gene trees and species trees sharing the same set of *k* taxa, our goal is to study the probability that a random gene tree set $\mathcal {G}$ containing *n* gene trees sampled under the multispecies coalescent model is a bipartition cover of a species tree *S*. We then use this calculation to set an upper bound on the number of loci *n* required so that with a specified minimum probability, a random *n*-locus gene tree set is a bipartition cover of *S*.

### Exact computation for four-taxon species trees

We first calculate for four-taxon species trees the exact probability that a gene tree set is a bipartition cover of a species tree. A four-taxon species tree *S* has only one nontrivial bipartition (Fig. 1), which appears in five of the 15 rooted gene tree topologies. Consider a species tree whose nontrivial bipartition is AB |CD. This bipartition appears in the gene trees with topologies ((AB),(CD)), (((AB),C),D), (((AB),D),C), (((CD),A),B), and (((CD),B,A)).

We compute the probability that a set $\mathcal {G}$ of gene trees is a bipartition cover for a four-taxon species tree *S* with bipartition AB |CD. Because the species tree has only one nontrivial bipartition, all that is required is for one of the gene trees in $\mathcal {G}$ to have one of the five topologies with the bipartition AB |CD. For four-taxon species trees, it is straightforward to calculate the probabilities under the multispecies coalescent model of each of the 15 gene tree topologies [27]. The probability that a gene tree possesses the species tree bipartition and hence is a bipartition cover is the sum of the probabilities of the five gene tree topologies with bipartition AB |CD.

**Fig. 2:**
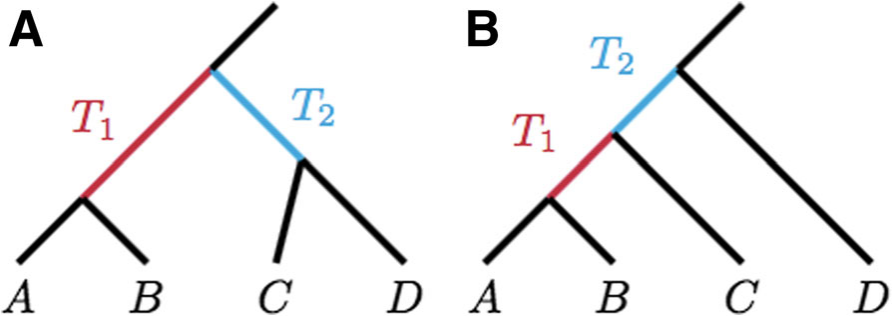
Two species tree topologies with four taxa. **a** Symmetric topology. **b** Asymmetric topology. Times *T*_1_ and *T*_2_ denote the species tree internal branch lengths

We must consider two cases, in which *S* represents the symmetric (Fig. 2 a) or asymmetric species tree topology (Fig. 2 b). Employing tabulations of gene tree probabilities for four-taxon species trees ([27], Tables 4 and 5), we examine both species tree topologies, denoting the probability that a gene tree has bipartition AB |CD in the symmetric case by ${P^{s}_{1}}$ and in the asymmetric case by ${P^{a}_{1}}$. The subscript 1 indicates that this quantity is for a single gene tree; we will generalize to sets of *n* gene trees in the next step. Labeling the species tree branch lengths in coalescent time units by *T*_1_ and *T*_2_ as in Fig. 2, in the symmetric case,

$$\begin{aligned} {P_{1}^{s}} = \ & \left[ g_{2,1}(T_{1})g_{2,1}(T_{2}) + \frac{1}{3}g_{2,1}(T_{1})g_{2,2}(T_{2}) \right. \\ \ & + \left. \frac{1}{3}g_{2,2}(T_{1})g_{2,1}(T_{2}) + \frac{1}{9}g_{2,2}(T_{1})g_{2,2}(T_{2}) \right] \\ \ & + \left[\frac{1}{3}g_{2,1}(T_{1})g_{2,2}(T_{2}) + \frac{1}{18}g_{2,2}(T_{1})g_{2,2}(T_{2})\right] \\ \ & + \left[\frac{1}{3}g_{2,1}(T_{1})g_{2,2}(T_{2}) + \frac{1}{18}g_{2,2}(T_{1})g_{2,2}(T_{2})\right] \\ \ & + \left[\frac{1}{3}g_{2,2}(T_{1})g_{2,1}(T_{2}) + \frac{1}{18}g_{2,2}(T_{1})g_{2,2}(T_{2})\right] \\ \ & + \left[\frac{1}{3}g_{2,2}(T_{1})g_{2,1}(T_{2}) + \frac{1}{18}g_{2,2}(T_{1})g_{2,2}(T_{2})\right]. \\ \end{aligned} $$

For the asymmetric case,

$$\begin{aligned} {P_{1}^{a}} = \ & \left[ \frac{1}{3}g_{2,1}(T_{1})g_{2,2}(T_{2}) + \frac{1}{9}g_{2,2}(T_{1})g_{3,2}(T_{2}) \right. \\ \ & + \left. \frac{1}{9}g_{2,2}(T_{1})g_{3,3}(T_{2}) \right] \\ \ & + \left[ g_{2,1}(T_{1})g_{2,1}(T_{2}) + \frac{1}{3}g_{2,1}(T_{1})g_{2,2}(T_{2}) \right. \\ \ & + \frac{1}{3}g_{2,2}(T_{1})g_{3,1}(T_{2}) + \frac{1}{9}g_{2,2}(T_{1})g_{3,2}(T_{2}) \\ \ & \left. + \frac{1}{18}g_{2,2}(T_{1})g_{3,3}(T_{2})\right] \\ \ & + \left[ \frac{1}{3}g_{2,1}(T_{1})g_{2,2}(T_{2}) + \frac{1}{9}g_{2,2}(T_{1})g_{3,2}(T_{2})\right.\\ \ & \left. +\frac{1}{18}g_{2,2}(T_{1})g_{3,3}(T_{2})\right] \\ \ & + \left[ \frac{1}{18}g_{2,2}(T_{1})g_{3,3}(T_{2}) \right] + \left[ \frac{1}{18}g_{2,2}(T_{1})g_{3,3}(T_{2}) \right]. \\ \end{aligned} $$

The five terms demarcated by brackets in ${P_{1}^{s}}$ and ${P_{1}^{a}}$ give the probabilities of the five gene tree topologies with bipartition AB |CD: ((AB),(CD)), (((AB),C),D), (((AB),D),C), (((CD),A),B), and (((CD),B,A)), respectively.

Simplifying these equations using Eq. 1, we find that

2 $$\begin{array}{@{}rcl@{}} {P_{1}^{s}} & = & 1-\frac{2}{3}e^{-(T_{1}+T_{2})} \end{array} $$

3 $$\begin{array}{@{}rcl@{}}  {P_{1}^{a}} & = & 1-\frac{2}{3}e^{-T_{1}}. \end{array} $$

Note that these two equations are similar in that in each case, the quantity in the exponent, *T*_1_+*T*_2_ or *T*_1_, corresponds to the length of the only internal branch of the unrooted species tree (Fig. 2).

Equations 2 and 3 give the probabilities that a single gene tree is a bipartition cover of the species tree, in the symmetric and asymmetric cases, respectively. Recall that our goal is to calculate the probability that a set $\mathcal {G}$ of *n* gene trees is a bipartition cover, or that the species tree bipartition is observed in at least one of *n* sampled gene trees. This quantity—${P_{n}^{s}}$ in the symmetric case and ${P_{n}^{a}}$ in the asymmetric case—is 1 minus the probability that the bipartition is observed in none of the *n* trees. Because each gene tree is independent conditional on the species tree, we have

4 $$\begin{array}{@{}rcl@{}} {P_{n}^{s}} & = & 1 - (1-{P^{s}_{1}})^{n} \end{array} $$

5 $$\begin{array}{@{}rcl@{}}  {P_{n}^{a}} & = & 1 - (1-{P^{a}_{1}})^{n}. \end{array} $$

**Fig. 3:**
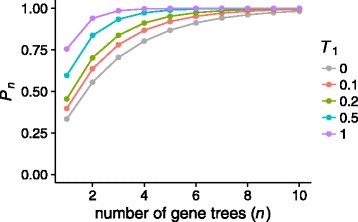
The probability (*P*_*n*_) that a random set of *n* gene trees under the multispecies coalescent is a bipartition cover of a four-taxon asymmetric species tree, as a function of *n*. Points represent the exact probability computed at each *n*, for several values of *T*_1_ (Eq. 5)

In Fig. 3, we plot ${P_{n}^{a}}$ as a function of the number of loci *n* for several fixed values of *T*_1_; the behavior of ${P_{n}^{s}}$ is analogous, except with *T*_1_ replaced by *T*_1_+*T*_2_. For each value of *T*_1_, ${P_{n}^{a}}$ increases with *n*, approaching 1 as *n*→*∞*. For larger *T*_1_, the initial probability that a single gene tree has bipartition AB |CD is greater, so that the number of gene trees required before ${P_{n}^{a}}$ achieves a specified value is smaller. As *T*_1_→0, gene trees approach a scenario in which the gene lineages from species A, B, and C persist into the common ancestor of the three species. Each possible sequence of coalescences among these three lineages is equally likely, and the probability that a random gene tree contains the nontrivial bipartition AB |CD is ${P_{1}^{a}} = \frac {1}{3}$. *P*_*n*_ then approaches $1-(\frac {2}{3})^{n}$.

### A general upper bound for *k*-taxon species trees

For *k*>4, the number of nontrivial bipartitions in a *k*-taxon species tree exceeds 1, and the event that a random gene tree possesses a nontrivial species tree bipartition *ϕ*_1_ is not independent of the event of its possessing a second such bipartition *ϕ*_2_. To perform a comparably simple calculation in the general *k*-taxon case to that achieved in the four-taxon case, we focus on deriving a lower bound on the probability that a random *n*-locus gene tree set $\mathcal {G}$ is a bipartition cover of a *k*-taxon species tree *S*.

Let *S* be a rooted *k*-taxon species tree with fixed topology and branch lengths. Denote the *k*−3 nontrivial bipartitions of *S* by *ϕ*_1_,*ϕ*_2_,…,*ϕ*_*k*−3_. Denote the *k*−2 internal branches of *S* by *e*_1_,*e*_2_,…,*e*_*k*−2_, with associated lengths *T*_1_,*T*_2_,…,*T*_*k*−2_. If one side of the root of *S* has only a single leaf, then the internal branch immediately descended from the other side is associated with a trivial bipartition. We indicate this internal branch by *e*_*c*_, with *c*∈{1,2,…,*k*−2}, and we denote its associated branch length *T*_*c*_. If both sides of the root of *S* each have at least two descendant leaves, then each of the *k*−2 internal branches is associated with a nontrivial bipartition, and the two branches immediately descended from the root share the same bipartition. We indicate by {1,2,…,*k*−2}∖*c* the set of indices for internal branches that produce nontrivial bipartitions, understanding that if the two sides of the root each have at least two descendant leaves so that *e*_*c*_ does not exist, this index set reduces to {1,2,…,*k*−2}.

Let *E*_*i*,*n*_ be the event that bipartition *ϕ*_*i*_ is observed at least once in a set $\mathcal {G}$ of *n* random gene trees, and let $Q_{i,n} = \mathbb {P}[E_{i,n}]$ be the associated probability that at least one of *n* random gene trees possesses *ϕ*_*i*_. Then *E*_*n*_=*E*_1,*n*_∩*E*_2,*n*_∩⋯∩*E*_*k*−3,*n*_ is the event that a gene tree set $\mathcal {G}$ with *n* gene trees is a bipartition cover of *S*. Denote by $Q_{n} = \mathbb {P}[E_{n}]$ the probability that a random gene tree set is a bipartition cover: that among *n* gene trees, all bipartitions of *S* appear at least once.

The *Q*_*i*,*n*_ have a complex dependence, so that if a gene tree possesses one of the bipartitions *ϕ*_*i*_, its conditional probability of possessing another bipartition *ϕ*_*j*_ might substantially increase in relation to the unconditional probability. Our strategy for bounding the desired probability *Q*_*n*_ from below amounts to supposing that each bipartition *ϕ*_*i*_ is as improbable as the least-probable bipartition and bounding the probability of the least-probable bipartition from below (Lemma 1). We then disregard the dependence among the *Q*_*i*,*n*_ to bound from below the joint probability that all of the *E*_*i*,*n*_ are observed in a gene tree set (Theorem 2).

Let *T*_min_= min*i*∈{1,2,…,*k*−2}*T*_*i*_ denote the length of the shortest internal branch in the species tree *S*. We obtain a lower bound on *Q*_*i*,*n*_, which we then use to bound *Q*_*n*_. Our lower bound for *Q*_*n*_ is a function of only *k*, *T*_min_, and *n*, and it can be inverted to produce an upper bound on the smallest *n* that achieves a desired minimal value for *Q*_*n*_.

#### **Lemma 1**

min*i*∈{1,2,…,*k*−3}*Q*_*i*,*n*_≥1−[1−*g*_*k*−2,1_(*T*_min_)]^*n*^.

#### *Proof*

Consider *Q*_*i*,*n*_ for some *i*. *Q*_*i*,*n*_ is the probability that bipartition *ϕ*_*i*_ is observed in at least one of *n* random gene trees that are conditionally independent given the species tree. It therefore equals 1 minus the probability that *ϕ*_*i*_ fails to be observed in all *n* gene trees: *Q*_*i*,*n*_=1−(1−*Q*_*i*,1_)^*n*^. Because for fixed *n*≥1, the function 1−(1−*x*)^*n*^ increases monotonically in *x* on [0,1],

6 $$\begin{array}{@{}rcl@{}}  \min_{i \in \{1, 2, \dots, k-3 \}} Q_{i,n} & = & \min_{i \in \{1, 2, \dots, k-3 \}} [ 1-(1-Q_{i,1})^{n} ]  \\ & = & 1- \left(1- \min_{i \in \{1, 2, \dots, k-3 \}} Q_{i,1} \right)^{n}. \end{array} $$

To produce a lower bound on min*i*∈{1,2,…,*k*−3}*Q*_*i*,*n*_, it remains to bound min*i*∈{1,2,…,*k*−3}*Q*_*i*,1_ from below. A sufficient condition for bipartition *ϕ*_*i*_ to be observed in a gene tree is for all the lineages descended from the internal branch $e_{\phi _{i}}$ associated with *ϕ*_*i*_ in the species tree to coalesce to a single lineage on that branch. In case *ϕ*_*i*_ is associated with two internal branches—the two immediately descended from the root on opposite sides—it is sufficient for the lineages on one side to coalesce to a single lineage on the internal branch associated with that side. Supposing that *k*_*i*_ is the number of taxa descended in *S* from branch *e*_*i*_ and *T*_*i*_ is the branch length for *e*_*i*_, the probability *Q*_*i*,1_ that *ϕ*_*i*_ is observed in a single gene tree is therefore bounded below by $\phantom {\dot {i}\!}g_{k_{i,1}}(T_{i})$, and:

7 $$ \begin{aligned} 1~- \ & \left(1- \min_{i \in \{1, 2, \dots, k-3 \}} Q_{i,1} \right)^{n} \\ \ & \geq 1- \left[ 1-\min_{i \in \{1, 2, \dots, k-2 \} \setminus c} g_{k_{i},1}(T_{i}) \right]^{n}. \end{aligned}  $$

In this step, although the species tree has *k*−3 nontrivial bipartitions, it has *k*−2 internal branches, one of which possibly produces a trivial bipartition. If cuts on two of the *k*−2 internal branches, say *j*_1_ with $k_{j_{1}}$ descendant leaf nodes and *j*_2_ with $\phantom {\dot {i}\!}k_{j_{2}}$ descendant leaf nodes, produce the same (nontrivial) bipartition *ϕ*_*i*_, then $\phantom {\dot {i}\!}Q_{i,1} \geq g_{k_{j_{1}},1}(T_{j_{1}})$ and $Q_{i,1} \geq g_{k_{j_{2}},1}(T_{j_{2}})\phantom {\dot {i}\!}$.

The quantity $g_{k_{i},1}(T_{i})\phantom {\dot {i}\!}$—the probability that *k*_*i*_ lineages coalesce to 1 lineage during time *T*_*i*_—decreases monotonically with increasing *k*_*i*_, and increases monotonically with increasing *T*_*i*_. Because a species tree internal branch associated with a nontrivial bipartition has at most *k*−2 descendant leaves, and because the shortest internal branch length is *T*_min_,

8 $$ g_{k_{i},1}(T_{i}) \geq g_{k-2,1}(T_{i}) \geq g_{k-2,1}(T_{\min}).  $$

This condition applies to each of the *k*−2 internal branches—including both immediately descended from the root in the case that the root does not have a pendant edge as one of its descendants. We take the minimum over internal branches that produce nontrivial bipartitions to obtain

9 $$ \min_{i \in \{1, 2, \dots, k-2 \} \setminus c} g_{k_{i},1}(T_{i}) \geq g_{k-2,1}(T_{\min}).  $$

We can connect inequalities 6, 7, and 9 to conclude

10 $$ \min_{i \in \{1, 2, \dots, k-3 \}} Q_{i,n} \geq 1- [ 1 - g_{k-2,1}(T_{\min})]^{n}.  $$

We thus have the desired result. □

The approach of this proof amounts to replacing the species tree *S* with $S_{T_{\min }}$, a tree with the same topology as *S* but with all internal branch lengths set to *T*_min_, the minimum branch length in *S*. Next, it is noted that each bipartition is at least as probable as the least probable bipartition. The probability of the least probable bipartition is then bounded from below by computing a lower bound on one specific way of observing an arbitrary bipartition: the probability of a bipartition is at least as great as the probability that all of the lineages for leaves that descend from its associated internal edge coalesce on that edge.

Now that we have a lower bound for the probability of an arbitrary bipartition, it remains to simultaneously consider all *k*−3 bipartitions.

#### **Theorem 2**

*Q*_*n*_≥1−(*k*−3)[1−*g*_*k*−2,1_(*T*_min_)]^*n*^.

#### *Proof*

As the probability of an intersection, *Q*_*n*_ can be written $Q_{n} = \mathbb {P}[E_{n}] = \mathbb {P}[\bigcap _{i=1}^{k-3} E_{i,n}]$. The minimal probability of the intersection of a set of possibly dependent events can be bounded by Bonferroni’s inequality [31]. It follows that

11 $$ Q_{n} \geq 1 - \sum_{i=1}^{k-3} \mathbb{P}[\overline{E}_{i,n}],  $$

where $\overline {E}_{i,n}$ is the complement of event *E*_*i*,*n*_.

We then have

12 $$ \begin{aligned} Q_{n} & \geq 1 - \sum_{i=1}^{k-3} (1 - \mathbb{P}[E_{i,n}]) \\ & = \left(\sum_{i=1}^{k-3} Q_{i,n} \right)- \left(k-4\right) \\ & \geq \left(k-3\right)\left(\min_{i \in \{1, 2, \dots, k-3 \} }Q_{i,n}\right) - (k-4). \\ \end{aligned}  $$

We invoke Lemma 1 to obtain min*i*∈{1,2,…,*k*−3}*Q*_*i*,*n*_≥1−[1−*g*_*k*−2,1_(*T*_min_)]^*n*^, from which

13 $$ Q_{n} \geq 1 - (k-3)[1 - g_{k-2,1}(T_{\min})]^{n}.  $$

This completes the proof. □

Note that given the species tree *S*, for small values of *n*, it is possible for (*k*−3)[1−*g*_*k*−2,1_(*T*_min_)]^*n*^≥1, so that the theorem produces a negative value for the lower bound on *Q*_*n*_. Because *Q*_*n*_ is a probability, in these cases, we have the trivial result *Q*_*n*_≥0. As *n* increases, however, eventually (*k*−3)[1−*g*_*k*−2,1_(*T*_min_)]^*n*^<1, so that in the theorem, *Q*_*n*_ is bounded from below by a positive quantity.

By solving for *n*, for a specified probability *q*, Eq. 13 can be used to calculate an upper bound on the minimal value of *n* for which *Q*_*n*_≥*q*. Setting *Q*_*n*_=*q* for 0<*q*<1,

14 $$ n = \frac{\log [(1-q)/(k-3)]}{ \log [1 - g_{k-2,1}(T_{\min})]}.  $$

Equation 14 gives an upper bound on the number of sampled gene trees required for a random gene tree set to be a bipartition cover with probability at least *q*. It applies irrespective of the species tree topology and branch lengths.

### Influences on the upper bound

**Fig. 4:**
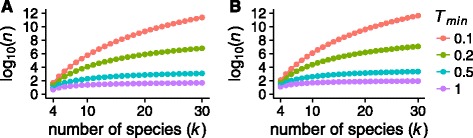
Upper bound on the number of gene trees required for a random set of *n* gene trees to have probability at least *q* of being a bipartition cover of a *k*-taxon species tree with smallest internal branch length *T*_min_. The plot uses Eq. 14. **a** *q*=0.99. **b** *q*=0.99999. The maximal number of independent gene trees in a genome is on the order of 10^4^ to 10^5^

For fixed values of *q*, we numerically computed the number of gene trees *n* required for achieving *Q*_*n*_≥*q* in Eq. 14. In Fig. 4, we plot log10(*n*) as a function of the number of taxa *k* for a range of minimum branch lengths and *q*=1−10^−2^ and *q*=1−10^−5^.

When *T*_min_=1 or *T*_min_=0.5, so that the shortest internal branch length in the species tree has a value of 1 or 0.5 coalescent time units, *n* grows slowly as a function of *k* and remains less than 10^4^ for species trees containing up to 30 species. By contrast, when *T*_min_=0.2 or *T*_min_=0.1, species trees with up to *k*=8 taxa have *n*<10^4^, but the number of gene trees *n* grows rapidly and exceeds 10^4^ for larger *k*. The patterns are fairly insensitive to the value of *q*, as *q* contributes to Eq. 14 only via the logarithmic term log(1−*q*).

### Accuracy of the upper bound

We next compared our upper bound on the number of loci required to produce a bipartition cover with probability *q* (Eq. 14) to values of this number of loci obtained in stochastic simulations under the multispecies coalescent. The simulations allow us to quantify the extent to which our upper bound overestimates the true number of required gene trees.

Simulations were conducted using COAL [25] to compute the exact multinomial distribution of gene tree topologies for “caterpillar” species trees in which all branch lengths were set to *T*_min_. The caterpillar case represents a difficult scenario for species tree inference, as the extent of gene tree discordance can be greater with caterpillar species trees than other species tree topologies [28, 29, 32, 33]. For fixed values of *n*_*s*_, the number of simulated gene trees in gene tree sets, we resampled 10^4^ independent gene tree sets from this exact multinomial distribution, identifying for each gene tree set all gene tree clades that appeared in at least one of the random gene trees. This clade identification step was conducted using Biopython [34].

Next, we recorded the empirical proportion of simulations in which the *n*_*s*_ gene trees produced a bipartition cover of the species tree. Treating this empirical probability of a bipartition cover as an estimate of $Q_{n_{s}}$, we then computed the number of loci *n* in Eq. 14 using the estimated $\hat {Q}_{n_{s}}$ for *q*, denoting this number of loci *n*_*b*_. The ratio $\frac {n_{b}}{n_{s}}$ represents the factor by which our upper bound on the minimum number of loci required for producing a bipartition cover exceeded the actual number of loci required in simulated gene tree sets. A value of $\frac {n_{b}}{n_{s}}=1$ indicates that our upper bound is accurate; values larger than 1 indicate that our upper bound overestimates the number of required gene trees by a factor of $\frac {n_{b}}{n_{s}}$.

**Fig. 5:**
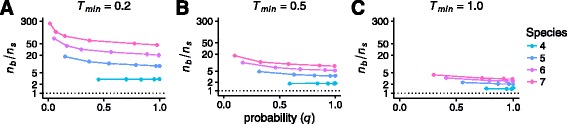
The ratio $\frac {n_{b}}{n_{s}}$ of the upper bound on the minimum number of gene trees required to obtain a bipartition cover with probability *q* (Eq. 14) to the corresponding number of simulated gene trees required to obtain a bipartition cover with probability *q*. The ratio is plotted as a function of *q*, for several values of the number of species *k*. **a** *T*_min_=0.2. **b** *T*_min_=0.5. **c** *T*_min_=1.0. The y-axis is plotted on a logarithmic scale. Irregular spacing of *q* values is a result of our simulation procedure, in which each *q* is determined from 10^4^ simulations at a fixed *n*_*s*_ in the set {1,2,3,5,10,20,50,100,200,500}. Note that for some large values of *n*_*s*_ at a fixed *T*_min_, all 10^4^ simulations produced a bipartition cover, meaning that $\hat {Q}_{n_{s}}=q=1$. In these cases, *n*_*b*_ computed from Eq. 14 is infinite and we do not plot $\frac {n_{b}}{n_{s}}$

Figure 5 presents $\frac {n_{b}}{n_{s}}$ as a function of *q*. In each panel, representing different values of *T*_min_, $\frac {n_{b}}{n_{s}}$ is relatively close to 1 for *k*=4 taxa, indicating a reasonably accurate upper bound. As *k* increases, $\frac {n_{b}}{n_{s}}$ progressively increases as well. For small *k*, with relatively few internal branches, fewer ways exist for coalescent events to occur other than on the internal branch of minimum length, so that our consideration of only those coalescences in obtaining the bound disregards fewer alternative ways of producing bipartitions. It hence produces a more accurate *n*_*b*_.

Comparing the three panels of Fig. 5, we see that $\frac {n_{b}}{n_{s}}$ is smaller and the bound *n*_*b*_ is therefore tighter when *T*_min_ is large than when *T*_min_ is small. For small *T*_min_, it is unlikely that all lineages below a species tree branch of length *T*_min_ will coalesce on the branch, so that our consideration of only the case in which such coalescences occur in producing Eq. 14 is less accurate. For each *T*_min_ value, the level of overestimation does not strongly depend on the value of *q*, especially for *q* near 1.

## Conclusions

We have derived a general analytical upper bound under the multispecies coalescent on the number of gene trees required for observing with a specified probability *q* all bipartitions of a species tree. In addition to the number of taxa and the probability *q*, our upper bound (Eq. 14) depends on a single parameter, the shortest internal branch of the true species tree. This simplicity enables general applicability of a bound that is relatively straightforward to calculate. We find that only a small number of gene trees is required, provided the minimum species tree branch length is not much shorter than the coalescent time scale ($T_{\min } \gtrsim 0.5$). Even when the shortest branch is small relative to the coalescent time scale (*T*_min_≈0.1), genomic studies of ≈ 10^4^ loci in *k*≲8 species will produce a bipartition cover of the species tree with high probability. Because our upper bound is a conservative overestimate, it is likely that the bipartition covers useful in the ASTRAL method [17, 18]—which relies on observing all bipartitions of the true species tree in a set of input gene trees—can often be achieved in realistic scenarios with considerably fewer loci.

### Species tree branch lengths

Because our upper bound depends on *T*_min_, to assess the number of gene trees required for producing bipartition covers in practical studies, we can examine the properties of *T*_min_ in models in which not only the gene trees are modeled conditional on fixed species trees, but in which the species trees are modeled as random quantities as well. Stadler & Steel ([35], Theorem 3.3) showed that in the Yule pure birth process for speciation, in which each species lineage speciates forward in time at rate *λ*, an arbitrary internal branch length has an exponential distribution with rate 2*λ*. The *k*−2 internal branch lengths in a species tree with *k* taxa are independent and identically distributed under the model. Hence, *T*_min_, as the minimum value of *k*−2 independent exponentially distributed random variables, each with rate 2*λ*, is exponentially distributed with rate $\sum _{i=1}^{k-2} 2\lambda = 2(k-2)\lambda $. The expected minimum species tree branch length under the Yule model is then $\mathbb {E}[T_{\min }]=1/[2(k-2)\lambda ]$.

To perform numerical calculations, we chose a range of values of *λ* on the basis of empirical studies; in the great apes, internal branch lengths of the species tree are consistent with a speciation rate of *λ*≈0.5 events per coalescent time unit [36, 37], and for primates, Stadler et al. [37] produced an estimate of *λ*≈0.28. In warblers, Bokma [38] estimated the rate of speciation to be 0.36 per million years. Assuming an effective population size of *N*_*e*_=5×10^4^ and a generation time of 1 year [39], we arrive at *λ*≈0.14 events per unit of time.

**Fig. 6:**
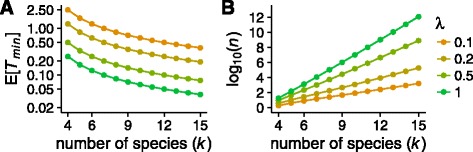
*T*_min_ under the Yule pure birth process for speciation at rate *λ* speciation events per coalescent time unit. **a** $\mathbb {E}[T_{\min }]$ as a function of the number of species *k*. The y-axis is plotted on a logarithmic scale. **b** The number of gene trees *n* required in Eq. 14 for obtaining with probability *q* all species tree bipartitions in a gene tree set, as a function of $\mathbb {E}[T_{\min }]$ values from **a**. The value of *q* is fixed at 0.99. Note that the maximal number of independent gene trees in a genome is approximately 10^4^ to 10^5^

In Fig. 6a, we plot $\mathbb {E}[T_{\min }]$ under the Yule model of speciation, as a function of the number of taxa *k* and the speciation rate *λ*.When speciation happens rarely relative to the coalescent timescale (*λ*≤0.2), for up to *k*=15 species, $\mathbb {E}[T_{\min }] \geq 1/(2 \times 13 \times 0.2) \approx 0.19$. When speciation events happen more frequently (*λ*=0.5), however, $\mathbb {E}[T_{\min }]$ goes below 0.19 at *k*=8 species, and $\mathbb {E}[T_{\min }] < 0.19$ for *k*=5 when *λ*=1.

Figure 6 b plots the value of *n* in Eq. 14 that is required to obtain a bipartition cover with probability *q*=0.99, as a function of the expected minimum branch lengths from Fig. 6 a. When speciation is slow (*λ*≤0.2, e.g. warblers), species trees with *k*=15 taxa achieve the high probability of 0.99 of producing bipartition covers with a number of gene trees comparable to the scale of the number of independent loci that might be present in a genome (*n*=10^4^ to 10^5^). With more frequent speciations, however (*λ*≥0.5), our upper bound on the required number of gene trees suggests an impractical number of gene trees. Recall that this scenario of large *k* and small *T*_min_ is precisely the case in which our upper bound is most conservative (Fig. 5), so that a stricter upper bound might indicate that the true required number of gene trees is in fact in a range that is practicable in principle.

### Extensions

Our analysis of the effect of the speciation rate *λ* on the number of gene trees required for observing a bipartition cover highlights both the utility and the limitations of our approach. The results apply irrespective of the number of species and the species tree topology and branch lengths; however, to obtain this generality, we have relied on approximations that make our bound conservative. To compute the probability that a gene tree set is a bipartition cover, in Lemma 1, we have assumed that each bipartition is only as probable as the least likely bipartition. Further, considering only the least likely bipartition has amounted to assuming that all branches have the same length as the shortest branch. We have also used a conservative lower bound for the probability of the least likely bipartition. In Theorem 2, we have conservatively assumed that the presence in a gene tree of one species tree bipartition does not affect the presence of another bipartition. By incorporating more parameters for the species tree rather than only the number of species and *T*_min_, each of these assumptions can potentially be relaxed to produce a more accurate upper bound on the number of gene trees required for obtaining a bipartition cover.

For example, consider our lower bound for the probability of the least likely bipartition, which assumes that *k*−2 lineages coalesce to a single lineage on the shortest species tree internal branch. Most species trees have no internal branch from which *k*−2 species descend; further, it is unlikely that if such a branch does exist that it is the shortest internal branch. Even in this scenario, many ways exist for the bipartition to be realized by a gene tree other than by all *k*−2 lineages coalescing on the shortest branch.

With the species tree branch lengths and topology taken into account, we can in fact calculate the probability of the least likely bipartition. Suppose a bipartition *ϕ* of the species tree separates the *k* taxa into two species groups, $\mathcal {T}_{\phi }$ and $\overline {\mathcal {T}}_{\phi }$. The probability that bipartition *ϕ* is observed in a gene tree is then the same as the probability that the gene lineages of the species in either $\mathcal {T}_{\phi }$ or $\overline {\mathcal {T}}_{\phi }$ (or both) are monophyletic:

15 $$ \mathbb{P}[E_{\phi,1}] = P_{M}(\mathcal{T}_{\phi}) + P_{M}(\overline{\mathcal{T}}_{\phi}) - P_{RM}(\mathcal{T}_{\phi},\overline{\mathcal{T}}_{\phi}),  $$

where *P*_*M*_ is the probability of monophyly of a set of gene lineages, *P*_*RM*_ is the probability of reciprocal monophyly of a pair of sets of gene lineages, and $\mathbb {P}[E_{\phi,1}]$ is the probability that the bipartition *ϕ* is observed in a random gene tree (by abuse of notation, we identify the gene lineages of species set $\mathcal {T}_{\phi }$ with $\mathcal {T}_{\phi }$, and similarly for $\overline {\mathcal {T}}_{\phi }$). Recently, Mehta et al. [40] derived formulas for *P*_*M*_ and *P*_*RM*_ for arbitrary gene lineage sets conditional on arbitrary fixed species trees with topology and branch lengths specified; using these formulas, it would be possible to exactly calculate the probabilities of each of the *k*−3 bipartitions, and to replace our lower bound on the probability of the least likely bipartition in Lemma 1 with the *exact* minimum.

We note than in addition to ASTRAL, other methods (including in problems with gene duplication and loss rather than incomplete lineage sorting [41]) employ similar constrained search algorithms relying on bipartitions. Some methods have the property that if the input gene tree set is a bipartition cover of the species tree, the true species tree lies in the search space and is feasible to produce as an estimate [12, 42]. Our work thus provides guidance on the maximum number of loci required before the true species tree enters the search space. As a calculation applicable to arbitrary species trees, considering single features and then examining their joint probability by use of a Bonferroni inequality, our approach might thus be applicable in other problems that require a lower bound on the probability that a property is achieved by a gene tree set, or an upper bound on the number of gene trees required for achieving the property. Though it disregards detailed information that might be available about the species tree, the generality of the approach has potential to provide useful bounds on probabilities that are otherwise difficult to evaluate.

## Methods

The methods are described throughout the Results and discussion section.
